# Effect of Thickness on the Uniaxial Compression Failure Behavior of CFRP Laminates

**DOI:** 10.3390/polym17182518

**Published:** 2025-09-17

**Authors:** Zixing Qin, Huiming Ding, Shiyang Zhu, Can Jin, Jian Wang, Jiaxin Li, Han Wang

**Affiliations:** 1 School of Mechanical Engineering, Zhejiang University, Hangzhou 310027, China; 2 College of Energy Engineering, Zhejiang University, Hangzhou 310027, China; 3 Donghai Laboratory, Zhoushan 316021, China; 4 Wuhan Second Ship Design and Research Institute, Wuhan 430205, China; 5 Jilin Guoxing Composite Material Co., Ltd., Jilin 132000, China

**Keywords:** carbon fiber composites, compression-resistant structures, thick laminate, compression properties

## Abstract

Carbon Fiber Reinforced Composite (CFRP) is widely used in deep-sea pressure-resistant structures. With the increase in submergence depth demand leading to the increase in the thickness of the CFRP shell plate, there is a significant thickness effect on its compression performance. In order to study the mechanism of the decrease in compression performance of the laminate, uniaxial compression tests, interlaminar shear tests, out-of-plane tensile tests, damage characterization, and FEM analysis were carried out on three thicknesses of laminates. The results showed that the compressive strength, interlaminar shear strength, out-of-plane tensile strength of laminates and FEM compression model decreased by 10.3%, 12.7%, 23.6%, and 13.6% when the thickness of the laminate was increased from 2 mm to 12 mm. Concurrently, the compression failure mechanism is transformed from the overall strength failure to the instability–crush failure mode caused by the initial delamination. The effects of out-of-plane tensile strength and interlaminar shear strength on compressive properties were also considered. It provides support for the regulation of compression performance of large-thickness laminates and the safety of deep-sea pressure-resistant structures in service.

## 1. Introduction

Carbon Fiber Reinforced Composites (CFRPs) exhibit high modulus, high specific strength, corrosion resistance, fatigue resistance, and superior impact resistance [1,2,3,4,5] and have become an important compositional approach to achieve light-weighting of deep-sea hull structures [6,7,8]. For instance, CFRP cylindrical shells have been implemented in subsea vessels such as autonomous underwater gliders (UG) [9], underwater vehicles (AUV) [10], human-occupied vehicles (HOV) [11,12], and composite pipelines [13]. However, as deep-sea hull equipment evolves toward larger dimensions and greater diving depths to meet extreme marine environmental demands, the external pressure-bearing capacity of CFRP hull structures faces heightened requirements. Consequently, the thickness of CFRP pressure-resistant shells must increase. However, there is a thickness effect in CFRP laminate. Compared to thin laminates, thick laminates tend to fail at lower stress levels and demonstrate reduced compressive strength [14,15,16,17]. This phenomenon underscores the necessity to scientifically characterize how thickness variation influences the compressive performance and failure mechanisms of CFRP. Such understanding is essential for ensuring the safe design of CFRP-based deep-sea hulls and other marine structural systems.

Current research usually defines laminate as thick laminate with a thickness of not less than 6 mm [18]. Gao et al. [19] conducted uniaxial compression tests on CFRP laminates with thicknesses of 40–70 mm and found that the compressive strength of the 70 mm thick laminate decreased by 10% compared to the 40 mm thick laminate, which showed a significant thickness effect related to the void defects and transverse shear effect within the laminate. The exponential empirical relationship between compression strength and thickness of CFRP laminates was established. Chen et al. [20] carried out uniaxial compression tests on 30 mm and 40 mm thick laminates, and the compression strength decreased by 6% after the increase in laminate thickness. Liu et al. [14] carried out relevant tests for three thicknesses of CFRP laminates, 3 mm, 6 mm, and 9 mm, and found that with the increase in thickness, the compressive strength of 6 mm and 9 mm laminates decreased by 7.05% and 10.31%, respectively. Hao et al. [21] conducted interlaminar shear tests on unidirectional glass fiber-reinforced epoxy composites with thicknesses ranging from 6 mm to 20 mm. Their results revealed a 15.7% reduction in interlaminar shear strength as thickness increased. The Weibull model was employed to quantitatively characterize the thickness-dependent dispersion of material properties. Gao [19] investigated ultra-thick CFRP laminates (30 mm) with different layers under uniaxial compression. The failure process exhibited a distinct “delamination followed by collapse” sequence, attributed to a 44% reduction in out-of-plane tensile strength compared to thinner counterparts. This degradation was further linked to the observed delamination behavior.

Due to the degradation of interlaminar properties, the failure characteristics of CFRP laminate under compressive loading shift with increasing thickness. Under compressive loading, intralaminar shear fracture occurs first in thin laminate, and shear cracks are mainly formed near the center layer, whereas the overall failure of thick laminate is caused by the premature delamination phenomenon, which leads to local instability [19,20]. However, Gao and Liu did not proceed to investigate the variation in interlayer properties with increasing thickness, nor did they use interlayer properties to explain the degradation of compressive properties with increasing thickness. Therefore, in order to deeply reveal the mechanism of compression strength reduction in CFRP thick laminate and the influence of interlaminar properties on this phenomenon, it is necessary to systematically investigate the correlation between compression strength and interlaminar shear strength and out-of-plane tensile strength of thick laminate, which is also a shortcoming of the current research.

To systematically explore the compressive failure mechanisms of thick CFRP laminates, this study integrates uniaxial compression tests, interlaminar shear tests, out-of-plane tensile tests, macro–micro damage characterization, and finite element method (FEM) analysis across three laminate thicknesses. The study innovatively establishes a correlation between macro-compressive strength and interlaminar properties by testing laminates of varying thicknesses. It confirms the shift in failure modes and evaluates the combined influence of interlaminar shear and out-of-plane tensile strengths on compressive capacity. Interpreting compressive performance based on interlayer properties not only reinforces the correlation derived from experiments but also highlights the critical role of interlayer properties in the compressive failure of thick laminates. These findings provide theoretical insights for optimizing the compressive performance of CFRP thick laminates and ensuring the operational safety of deep-sea pressure-resistant structures.

## 2. Experimental Methodologies

### 2.1. Specimen Preparation

**Figure 1:**
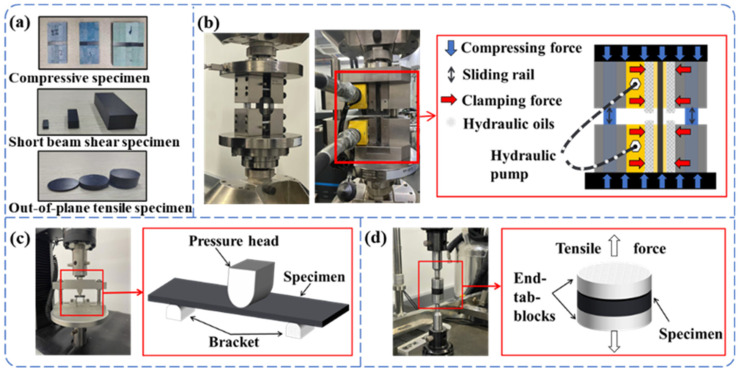
(**a**) Physical drawing of the sample to be tested. (**b**) Schematic diagram of the uniaxial compression test system. (**c**) Schematic diagram of the interlaminar shear test system. (**d**) Schematic diagram of the out-of-plane tensile test system.

In this study, carbon fiber/epoxy CFRP prepreg USN25000-7901 with a nominal thickness of 0.25 mm, a fiber volume fraction of 65 ± 2%, and a fiber volume fraction of 55% was provided by Weihai Guangwei Composites Co. (Weihai, China). Three different thicknesses (2 ± 0.1 mm, 6 ± 0.1 mm, and 12 ± 0.3 mm) of laminates were prepared using this prepreg, with quasi-isotropic layups for uniaxial compression and out-of-plane tensile specimens and unidirectional layups for interlaminar shear specimens (Figure 1a). The laminate was autoclave-cured under 0.6 MPa pressure with the following thermal cycle: heat from room temperature to 80 °C at a rate of 2 °C/min, maintain at 80 °C for 30 min, then heat to 120 °C at a rate of 2 °C/min, maintain at 120 °C for 60 min, and finally cool to room temperature at a controlled rate of 1.5 °C/min [14,19].

### 2.2. Test Methods

#### 2.2.1. Uniaxial Compression Test

At present, the compression performance test of CFRP thin laminate mainly refers to the standards ASTM D6641 [22], ASTM D3410 [23], GB/T 1448 [24]. The thickness of the laminate not less than 12 mm has exceeded the scope of application of the above standards, and it is necessary to design the compression specimen applicable to the laminate of 12 mm thickness. The compression specimen should have strength failure before the occurrence of Eulerian buckling, and the gauge section should not be too long [25]. The gauge section of the compression specimen should not be too short, and the edge effect of the reinforcing sheet should be taken into account to have a suitable stress uniformity zone. The final determination of the laminate compression specimen sizes for three thicknesses (Table 1) was made based on Equations (1)–(3) [14]. The reinforcing sheets at both ends were bonded to the test piece using Araldite 420A/B provided by Huntsman Advanced Chemicals Materials Ltd. (Guangzhou, China).

(1) $$\sigma_{c}\leq \frac{0.67k\pi^{2}\left(EI\right)}{AL^{2}}$$

(2) $$I=wt^{3}/12$$

(3) $$L_{max}\leq \pi t\sqrt{\frac{0.67kE}{12\sigma_{c}}}$$

where k is assumed to be 5/6 for laminate, $\sigma_{c}$ is the ultimate compressive strength, E is the Young’s modulus of the material, I is the moment of inertia of the section, A is the cross-sectional area of the specimen, L is the length of the gauge section, w is the width of the specimen cross-section, and t is the thickness of the specimen cross-section.

The compression tests were carried out using an IST ED25.305 (Yi Test Equipment Hangzhou Co., Ltd., Hangzhou, China) universal testing machine with a maximum load of 300 kN and a loading speed of 1.3 mm/min. The 2 mm and 6 mm thick laminate specimens were clamped in a typical combined loading compression (CLC) test fixture according to ASTM D6641. The 12 mm thick laminate was hydraulically driven to clamp the test piece, and the compression load was transferred to the specimen spacing section using a mixture of axial compression and shear loading (Figure 1).

#### 2.2.2. Interlaminar Shear Test

The interlaminar shear test was performed according to standard ASTM D2344 [26] and the interlaminar shear specimens are shown in Figure 1a. An INSTRON 34TM-50 (INSTRON CORPORATION, Boston, MA, USA) testing machine with a maximum load of 50 kN was used, equipped with a short beam shear test fixture. The loading rate was 1 mm/min and the test schematic is shown in Figure 1c.

#### 2.2.3. Out-of-Plane Tensile Test

The out-of-plane tensile test was performed according to standard ASTM D7291 [27], and the out-of-plane tensile specimen is shown in Figure 1a. An INSTRON 8500 (INSTRON CORPORATION, Boston, MA, USA) testing machine was used, with a maximum load of 100 kN and a loading rate of 0.1 mm/min. The out-of-plane tensile specimen was mounted between tensile blocks connected to the test fixture through a universal swivel joint, ensuring adaptive centering during loading, as schematically illustrated in Figure 1d.

### 2.3. Characterization Methods

This study employed a Sony Alpha 7RV camera (Sony Corporation, Tokyo, Japan) for macro-level damage analysis. Additionally, a Hitachi S-3400 scanning electron microscope (Hitachi Limited, Tokyo, Japan) and an Olympus CX53 microscope (Olympus Corporation, Tokyo, Japan) was utilized for micro-level damage analysis.

## 3. Results and Discussion

### 3.1. The Performance of Compression

**Figure 2:**
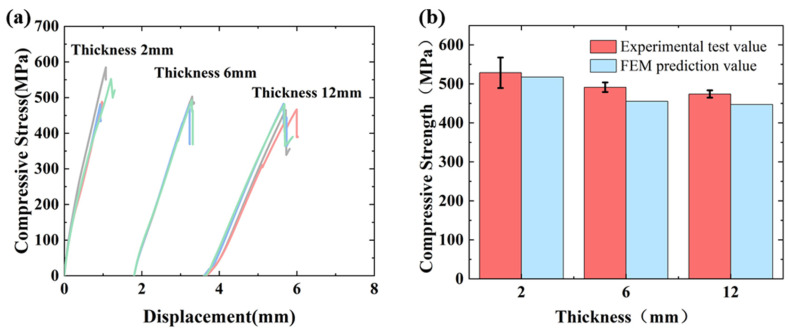
(**a**) Compression test stress–displacement. (**b**) Compression test result.

The CFRP compressive load–displacement curves are shown in Figure 2a, the curve shows a linear loading response in the early stage. With the occurrence of initial micro-buckling and interface failure, the stiffness of the local area will be reduced, leading to more buckling and damage. This cumulative damage process is manifested macroscopically as the generation of a nonlinear stress–strain curve. And after reaching the maximum load, the force decreases extremely rapidly. While the thick specimen surface produced an initial local delamination, a very slight decrease in the curve can be observed [20]. The compression test results are shown in Figure 2b. The dispersion of the ultimate strength of the same group of specimens is due to the influence of defects in the manufacturing process. The maximum coefficient of dispersion of the measured compression strength of the three thicknesses of laminate is 7.8%, which is not more than 10%, and the test results have a good consistency.

As the specimen thickness increased from 2 mm to 6 mm and 12 mm, the compressive strength decreased from 528.7 MPa to 491.4 MPa (7.1% reduction) and 474.1 MPa (10.3% reduction), respectively. This decline in compressive strength with increasing laminate thickness demonstrates a significant thickness effect on the compressive properties of CFRP. The results of this test further verified that the variation in the thickness is an important factor affecting the ultimate compressive strength [28], which verified the accuracy of the trend of the test results.

### 3.2. The Performance of Interlaminar Shear

**Figure 3:**
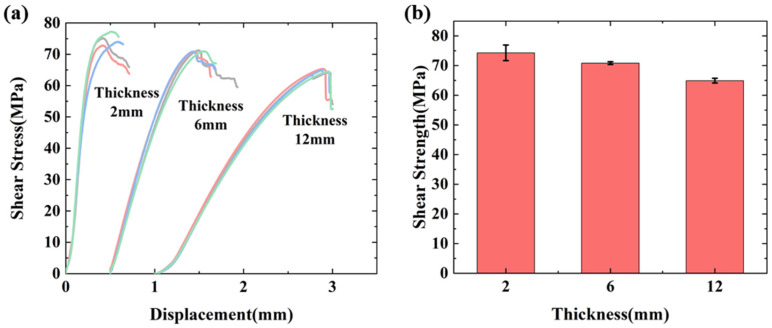
(**a**) Test stress–displacement of interlaminar shear test. (**b**) Test results of interlaminar shear test.

The measured shear stress–displacement curves and interlaminar shear strengths of CFRP are shown in Figure 3. The stress–strain curve initially exhibits linear elasticity, followed by progressive nonlinearity under increasing load. This nonlinear behavior becomes more pronounced with greater laminate thickness, attributed to enhanced interlayer deformation heterogeneity within composite layers. When cracking occurs in the middle section of the specimen, the curves will decrease sharply. With the increase in laminate thickness, the nonlinear characteristics are more significant, indicating that the process of interlaminar crack extension is nonlinear. The maximum dispersion coefficient of the measured interlaminar shear strength of the three thicknesses of laminate is 9.6%, which does not exceed 10%, and the test results are in good agreement. When the specimens were increased from 2 mm to 6 mm and 12 mm, the ultimate shear strength decreased from 74.3 MPa to 70.8 MPa and 64.9 MPa, with a decrease of 4.7% and 12.7%, respectively. The interlaminar strength of CFRP laminates also showed a decreasing trend as the laminate thickness increased, indicating that there was also a significant thickness effect on the interlaminar properties.

For the data in the interlaminar shear tests, the Weibull model can effectively explain the thickness effect. The Weibull model is usually used to explain the size effect, and the probability of sample-making defects in the specimen is taken as a controlling factor affecting the strength of interlaminar shear, and the relationship between ILSS (Interlaminar Shear Strength) and thickness between different specimens can be expressed as follows [29,30]:

(4) $$\frac{S_{1}}{S_{2}}={\left(\frac{V_{2}}{V_{1}}\right)}^{1/m}={\left(\frac{t_{2}}{t_{1}}\right)}^{3/m}$$

where $S_{1}$ and $S_{2}$, $V_{1}$ and $V_{2}$, $t_{1}$ and $t_{2}$ represent the interlaminar shear strength, specimen volume and thickness of the different specimens, respectively, and m is the Weibull modulus, which is used to characterize the size effect induced by the defects in the material. By logarithmic processing of the strength data of the three sets of specimens, m = 41 was calculated by the least-squares fitting method, which is close to the currently known results [21,31]. In particular, when the thickness of the specimen is increased from 2 mm to 6 mm, its ILSS is predicted to decrease by about 12.3%, according to Equation (4), which is close to the 12.7% strength decrease in the test. The coefficient of variation (COV) of strength was inferred from the Weibull modulus m:

(5) $$COV=\frac{1.2}{m}$$

The coefficient of variation of 2.90% calculated theoretically by Equation (5) is in high agreement with the measured value of 1.28% in the test. Therefore, the ILSS of specimens of different sizes can be predicted and estimated using the Weibull model, which provides theoretical support for the optimization of the thickness effect in the design of composite structures.

**Figure 4:**
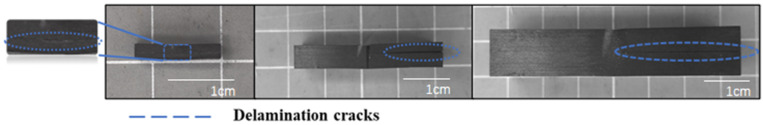
Failure diagram of interlaminar shear specimen.

Interlaminar shear strength testing revealed that all specimens failed via delamination, with the delamination propagation area exhibiting a thickness-dependent increase [32]: 0.16 cm^2^ (2 mm), 1.8 cm^2^ (6 mm), and 5.4 cm^2^ (12 mm) (Figure 4). The failure mode was interlaminar shear cracking and extended along the layer, so the interlaminar shear resistance was closely related to the interlaminar shear stresses. The cause of the weakening of the interlaminar shear capacity of the thicker specimen may be the defects that existed in the fabrication of the specimen [21].

### 3.3. The Performance of Out-of-Plane Tensile

**Figure 5:**
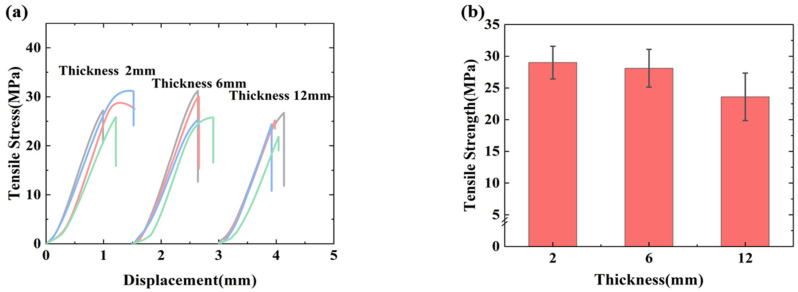
(**a**) Out-of-plane tensile test stress–displacement. (**b**) Out-of-plane tensile test results.

Figure 5a shows the stress–displacement diagram in the out-of-plane tensile test, where the specimen undergoes a linear mechanical response. As loading continues, minute defects within the specimen induce initial delamination, reducing the effective bearing area and causing a gradual decline in stiffness. This steady propagation of interlaminar damage leads to pronounced nonlinear behavior. Then it suddenly breaks with a sharp decrease in the curve. From Figure 5b, it can be seen that the out-of-plane tensile strength decreases slightly with the increase in specimen thickness, comparing the out-of-plane strength of 29.0 MPa for the 2 mm specimen with the out-of-plane strengths of 28.1 MPa and 23.6 MPa for the 6 mm and 12 mm specimens, which is a decrease of 3.1% and 18.6%, respectively. This occurs because composite laminates exhibit poor out-of-plane load resistance. Thicker laminates possess reduced interlaminar strength, making them susceptible to matrix cracking and delamination under out-of-plane tensile stresses. Consequently, increased thickness promotes premature failure and diminished out-of-plane tensile strength.

**Figure 6:**
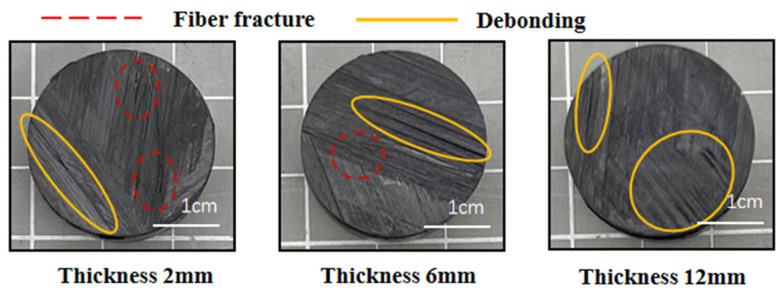
Sectional damage of out-of-plane tensile specimens of different thicknesses.

Comparative analysis of out-of-plane tensile fracture surfaces in CFRP laminates of varying thicknesses (Figure 6) revealed distinct failure mechanisms. The 2 mm specimen exhibited predominant fiber breakage with limited interlaminar debonding, whereas the 6 mm specimen displayed extensive interlaminar debonding accompanied by minor fiber breakage. The 12 mm specimen failed mainly through interlaminar debonding. Thus, with the increase in laminate thickness, the fracture failure mode changes from a mixed failure mode of fiber fracture and interlaminar debonding to an interlaminar debonding mode. This is because the thick specimen void content rises, and the crack fracture state is also due to the increase in void to produce stress concentration caused by the initial emergence of cracks [33,34]. Concurrently, reduced interlaminar strength accelerates premature interfacial failure under tensile loading. This degradation suppresses conditions for fiber-dominated fracture, resulting in interlaminar debonding as the primary failure mode. These findings conclusively demonstrate that increased laminate thickness exacerbates interlaminar bond deterioration, directly compromising structural performance.

### 3.4. Influence of Void Content

**Figure 7:**
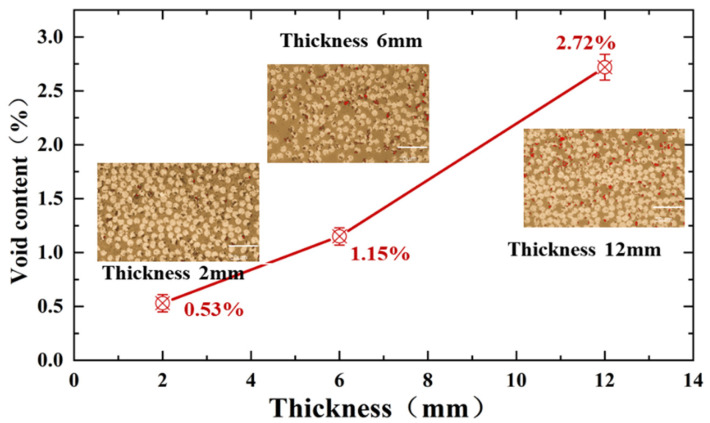
Void distribution of specimens of different thicknesses. scale bar = 20 μm.

In this study, three thickness specimens of 2 mm, 6 mm, and 12 mm were analyzed by microscopic quantitative analysis. As shown in Figure 7, as the specimen thickness increased from 2 mm to 6 mm and 12 mm, the void content increased significantly from 0.53% to 1.15% and 2.72%. The microstructures still showed significant differences: the fiber distribution of the 2 mm specimen was uniform and dense, while the 6 mm and 12 mm specimens showed more obvious resin-enriched zones and disordered fiber arrangement. This internal structural difference originates from the curing process of the thick plate, which may lead to uneven heat transfer during the curing process to produce a temperature gradient [35], resulting in differences in the mobility of the resin and the resin retention in the middle layer to form a migration barrier. The resin flow path is affected, and the resin in the central layer cannot be effectively migrated, which forms a resin-rich zone at the interface between the layers. Moreover, the stagnant resin in the central layer absorbed the gas generated during the curing process and inhibited the void migration [36]. The combined effect of the pore distribution and resin buildup affected the adjustment of the fiber position, resulting in the presence of continuous voids and unevenly distributed fibers in the middle layer of the thick laminate [37,38,39]. Therefore, specimens with the above defects are more prone to interfacial damage and evolve into delamination failure during the load-bearing process, which manifests itself in lower interlaminar shear properties and out-of-plane tensile properties.

## 4. Mechanism of Thickness Effect on Uniaxial Compression Performance

### 4.1. FEM Model Description

In this paper, the uniaxial compression finite element model of three thicknesses of laminate was established by using ABAQUS explicit module. The *X*-axis, *Y*-axis, and *Z*-axis correspond to the fiber direction, the specimen width direction, and the thickness direction of the laminates, respectively. The material properties are given in Table 2.

**Figure 8:**
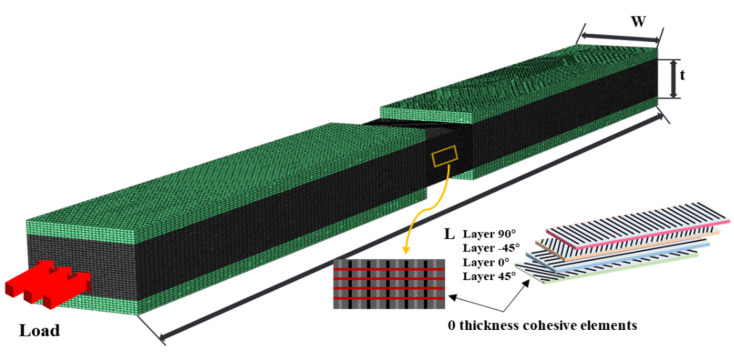
FEM model of laminate in compression.

The models employed 8-node C3D8R elements and zero-thickness Cohesive COH3D8 elements between layers to simulate the stiffness, strength and fracture toughness of the interlayer interfaces, and to capture the initiation and propagation of damage. One end of the model was subjected to fixed constraints via a reference point, while the other end was subjected to an axial displacement load through a reference point. Constraints in the thickness direction were applied to the surface of the reinforcement, which could undergo axial displacement during loading (Figure 8).

The intralaminar damage was assessed using the 3D Hashin criterion (Table 3) to determine the onset of damage. A stiffness degradation method based on fracture energy was used to simulate the progressive failure process [41]. The structural stiffness was reduced by introducing a damage state variable $D_{i}$ into the stiffness matrix. For each failure mode, the damage state variable was defined as $D_{i}$ = 0 at the onset of damage and D = 1 at complete failure (Equation (6)).

(6) $$D_{i}=max\left\{0,\;min\left\{1,\;\frac{\delta_{eq}^{f}\left(\delta_{eq}-\delta_{eq}^{0}\right)}{\delta_{eq}\left(\delta_{eq}^{f}-\delta_{eq}^{0}\right)}\right\}\right\}$$

where $\delta_{eq}^{0}$ is the equivalent strain in the starting state of composite failure, $\delta_{eq}^{f}$ is the equivalent strain in the complete failure state, and $\delta_{eq}$ is the equivalent strain in the current state.

### 4.2. Failure Mechanism Analysis

**Figure 9:**
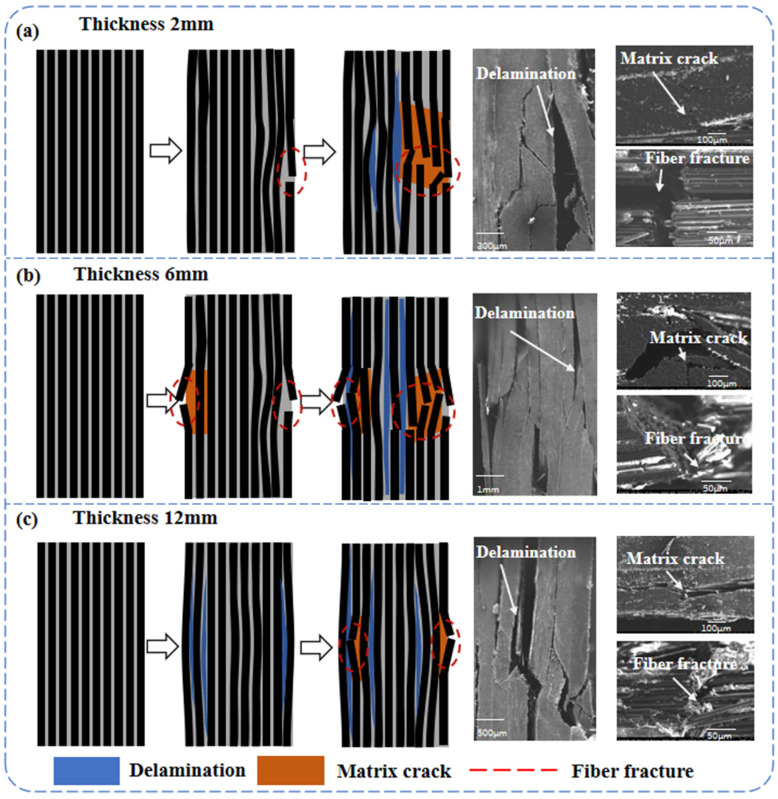
(**a**) Section of 2 mm thick compression specimen and SEM figure. (**b**) Section of 6 mm thick compression specimen and SEM figure. (**c**) Section of 12 mm thick compression specimen and SEM figure.

**Figure 10:**
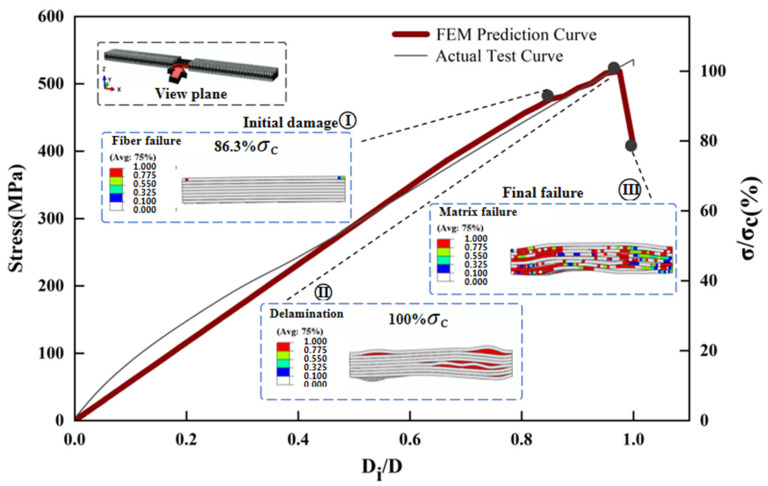
Finite element analysis of damage evolution of specimens with 2 mm laminate.

**Figure 11:**
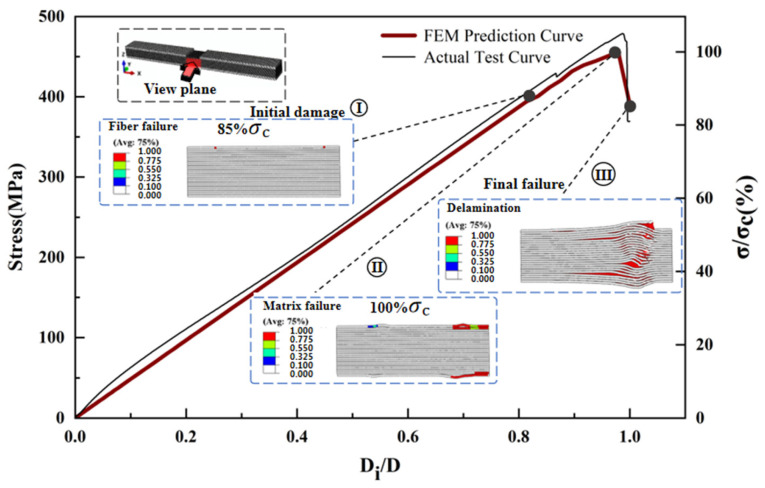
Finite element analysis of damage evolution of specimens with 6 mm laminate.

**Figure 12:**
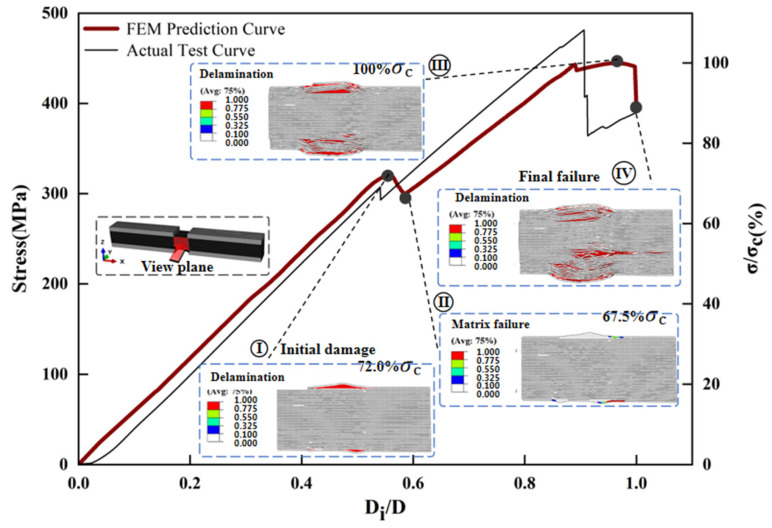
Finite element analysis of damage evolution of specimens with 12 mm laminate.

The maximum deviation between the predicted and measured compressive strengths of the three different thickness laminates compression models is 7.3% (Figure 2). Figure 9, Figure 10, Figure 11 and Figure 12 simulates the damage initiation and evolution processes in specimens of different thicknesses, revealing the thickness effect on the damage evolution patterns. Furthermore, Figure 10, Figure 11 and Figure 12 compare the experimental results curves with the FEM results curves, which demonstrate the rationality of the FEM model.

For the 2 mm specimen, when the load strength of the 2 mm specimen reached 86.3% of the ultimate strength, the initial compression damage occurred in the fibers of the surface layer of the specimen. At this stage, there were also some small fracture sounds during the test, but the structure of the specimen still had a good load-bearing capacity. As loading continued and the load strength approached the ultimate strength, local delamination developed in the surface layer, accompanied by slight fiber buckling and compression failure of the matrix. When the ultimate strength was reached, matrix cracking extended to the central layer region, accompanied by fiber failure, which caused delamination of the central layer of the specimen, leading to a final overall strength failure (Figure 9 and Figure 10).

For the 6 mm specimen, when the load strength reached 85% of the ultimate strength, the initial damage was manifested as compression damage of the surface fibers, and the damage gradually expanded towards the center. When the load strength reached 97.6% of the ultimate strength, the surface matrix reached compression failure, which is also accompanied by surface delamination. As the shear force S_13_ increased further, it led to more matrix failure around the fractured fibers, which in turn allowed the fibers to bend and fracture further [14]. At the ultimate load, the failure of the matrix caused delamination to quickly spread to the intermediate regions, eventually leading to compression failure with a mixture of delamination–shear damage between multiple layers (Figure 11).

From Section 3.2 and Section 3.3, the interlaminar shear strength and out-of-plane tensile strength of the thick laminate decreased by 12.7% and 18.6%, respectively, indicating weak interfacial strength [18]. Therefore, when the load strength reached 72.0% of the ultimate strength, the out-of-plane stress components S_33_ and S_13_ generated during compression led to earlier initial damage in the 12 mm specimen, which is manifested as localized delamination of the specimen surface. The curve produces a downward trend at this point and is accompanied by compression damage to the fibers. Delamination leads to a degradation of the specimen stiffness, as evidenced by a decrease in the slope of the load–displacement (Figure 12), while good load-bearing capacity persists at this stage. Continued loading was followed by damage to the matrix in the vicinity of the delamination region. At ultimate load, delamination propagates radially from the gauge section towards both specimen ends. This progressive damage renders the delaminated regions incapable of load transfer, accompanied by severe stiffness degradation. The decrease in the effective bearing thickness of the specimen results in a weaker resistance to instability, thus inducing a final instability–crush failure mode under high-pressure loading.

The results demonstrate a propensity for delamination initiation near the 0° ply, attributed to pronounced interlaminar stress heterogeneity under compressive loading, where axial stresses in the 0° ply significantly exceed those in adjacent plies. Under the action of out-of-plane stress components, this directly induces micro-buckling of fibers and delamination failure in the 0° ply during compression [14,45], leading to a final instability–crush failure mode.

**Figure 13:**
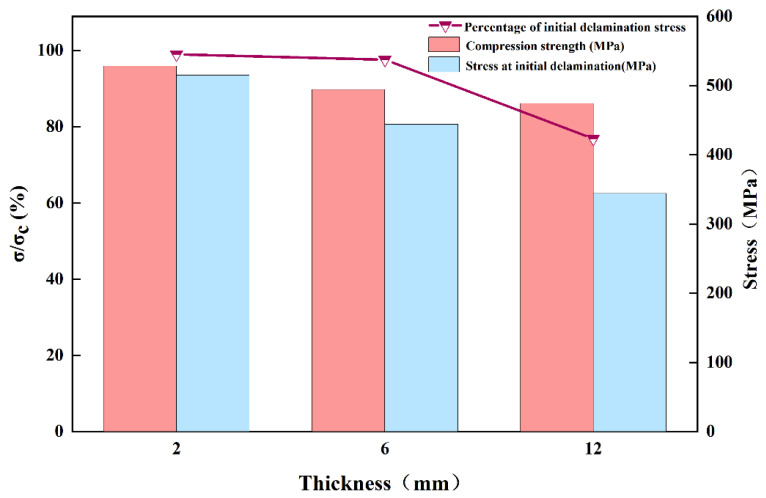
Initial delamination stresses and percentage for specimens of different thicknesses.

Therefore, as the thickness of the laminate increases, the compressive failure mechanism changes from the overall strength failure to the delamination-dominated crushing failure. This is due to the high internal void content of thick laminate, which inevitably generates stress concentration under compressive loading [46]. As mentioned in Section 3.2 and Section 3.3, increasing thickness leads to deterioration in interlaminar shear properties and out-of-plane tensile performance. When subjected to uniaxial compression, the stress components S_13_ (interlaminar shear stress) and S_33_ (out-of-plane tensile stress) cause premature damage in thicker specimens—including delamination, matrix cracking, and interfacial debonding—due to their weaker interlaminar characteristics. This results in reduced compressive performance and altered damage mechanisms, which macroscopically manifest as premature delamination (Figure 13). The important influence of the thickness effect on the failure mode of the specimens is confirmed by the above results.

## 5. Conclusions

In this study, uniaxial compression tests, interlaminar shear tests, out-of-plane tensile tests, damage characterization, and FEM analyses were carried out on three thicknesses of laminate to investigate the effects of thickness variation on compressive strength and interlaminar properties and the transformation of compressive failure forms, and the following conclusions were drawn:

Firstly, the void content of the laminates increased from 0.53% to 2.72% as the thickness increased from 2 mm to 12 mm. The compressive, interlaminar shear, and out-of-plane tensile properties decreased by 10.3%, 12.7%, and 18.6%, respectively, showing an obvious thickness effect.

Secondly, a FEM model considering intralaminar damage and interlaminar damage was developed, and the maximum deviation of 7.3% from the experimental values proved the reasonableness of the model.

Lastly, as the thickness increases from 2 mm to 12 mm, the interlaminar properties decrease and the compression failure mechanism is transformed from the overall strength failure to the final instability–crush failure mode caused by the initial delamination.

This study summarizes the effect of thickness on the interlaminar and compressive properties of quasi-isotropic laminates and explores the effect of thickness on the variation in compressive failure forms of laminates, which can provide an important theoretical basis and experimental reference for the design of composite structures.

## Figures and Tables

**Table 1:** CFRP compression test specimen size.

Laminate Thickness (t/mm)	Total Length (L/mm)	Gauge Length (L_0_/mm)	Width (w/mm)	Tab Thickness (t_1_/mm)
2	140	13	13	1.5
6	140	13	13	1.5
12	140	15	15	4

**Table 2:** Material properties of USN25000F-7901 [40].

**Intralaminar Properties**	$E_{11}$ = 120 GPa, $E_{22}$ = $E_{33}$ = 8 GPa, $G_{12}$ = $G_{13}$ = 4.5 GPa, $G_{23}$ = 3 GPa $\nu_{12}$ = $\nu_{13}$ = 0.25, $\nu_{23}$ = 0.3, ρ = 1600 (kg/m^3^); $X_{t}$ = 1600 MPa, $X_{c}$ = 1200 MPa, $Y_{t}$ = 55 MPa, $Y_{c}$ = 200 MPa $S_{12}$ = $S_{13}$ = 100 MPa, $S_{23}$ = 90 MPa;
**Interlaminar Properties**	$\tau_{n}$ = 35 MPa, $\tau_{s}$ = 60 MPa, $\tau_{t}$ = 60 MPa, $G_{1}^{c}$ = 0.6 N/mm, $G_{2}^{c}$ = $G_{3}^{c}$ = 2.1 N/mm;

**Table 3:** Three-dimensional Hashin failure criterion [42,43,44].

Failure Mode	Failure Criterion
Fiber tensile failure ($\sigma_{11}\geq 0$)	$F_{ft}={\left(\frac{\sigma_{11}}{X_{t}}\right)}^{2}+{\left(\frac{\sigma_{12}}{S_{12}}\right)}^{2}+{\left(\frac{\sigma_{13}}{S_{13}}\right)}^{2}\geq 1$
Fiber compressive failure ($\sigma_{11}<0$)	$F_{fC}={\left(\frac{\sigma_{11}}{X_{C}}\right)}^{2}\geq 1$
Matrix tensile failure ($\sigma_{22}+\sigma_{33}\geq 0$)	$F_{mt}={\left(\frac{\sigma_{22}+\sigma_{33}}{Y_{t}}\right)}^{2}+\left(\frac{{\sigma_{23}}^{2}-\sigma_{22}\sigma_{33}}{S_{23}}\right)+{\left(\frac{\sigma_{12}}{S_{12}}\right)}^{2}+{\left(\frac{\sigma_{13}}{S_{13}}\right)}^{2}\geq 1$
Matrix compressive failure ($\sigma_{22}+\sigma_{33}<0$)	$F_{mc}=\frac{\sigma_{22}+\sigma_{33}}{Y_{c}}\left[{\left(\frac{Y_{c}}{2S_{23}}\right)}^{2}-1\right]+\left(\frac{{\sigma_{23}}^{2}-\sigma_{22}\sigma_{33}}{S_{23}}\right)+{\left(\frac{\sigma_{22}+\sigma_{33}}{2S_{23}}\right)}^{2}+{\left(\frac{\sigma_{12}}{S_{12}}\right)}^{2}+{\left(\frac{\sigma_{13}}{S_{13}}\right)}^{2}\geq 1$

## Data Availability

Data sets generated during the current study are available from the corresponding author on reasonable request.

## Abbreviations

CFRP	Carbon Fiber Reinforced Composite
ILSS	Interlaminar Shear Strength
